# A Case Study of Anorexia Nervosa Driven by Religious Sacrifice

**DOI:** 10.1155/2014/512764

**Published:** 2014-07-06

**Authors:** Amelia A. Davis, Mathew Nguyen

**Affiliations:** University of Florida, 4037 NW 86 Terrace, Gainesville, FL 32606, USA

## Abstract

Anorexia nervosa (AN) is considered a relatively “modern” disorder; however, a number of scholarly works have cited examples of voluntary self-starvation dating back to several centuries. In particular, there are many examples of female starvation for religious reasons during the medieval period, with many being elevated to sainthood. We present a case of an elderly woman with AN who began restricting her diet when she was 13-years old while studying to be a nun at a Catholic convent. She reports that, during the development of her disease, she had no mirrors and, rather than restricting her diet to be thin or attractive, she restricted her diet to be closer to God in hopes of becoming a Saint. This unique case presents an opportunity to deepen our understanding of AN and the cultural context that affects its development.

## 1. Introduction

Anorexia nervosa (AN) is a syndrome that is more prevalent in industrialized and western cultures; it is more prevalent among females than males and has a peak age of onset during adolescence [1]. AN appears to have gained more popularity and professional attention over recent decades during a cultural period that idealizes thinness, with magazines publishing significantly more articles on methods for weight loss [2, 3]. Patients with AN are characterized by a disturbed body image in which they often have an intense preoccupation with weight, an intense fear of gaining weight despite having significantly low body weight, or persistent behavior that interferes with weight gain [4]. While once considered a cultural-bound syndrome that occurs almost exclusively in western cultures, a recent review of eating disorders in a cross-cultural and historical context indicates that AN is not a cultural-bound syndrome, although certain features of the disorder appear to be culturally bound, such as fear of weight gain or of becoming fat [5, 6]. AN may take different forms in different cultural and historical contexts with one unifying theme of morbid self-starvation. While previously characterized as a disorder that only occurs in the western cultural idealization of thinness and pressures to lose weight, a review of the literature suggests a long-standing relation between self-starvation and religious asceticism [7–10].

We describe below a contemporary case of an individual whom we treated for AN who developed her eating disorder at the age of 13 while living in a Catholic nunnery, in an environment with few mirrors and without pressure to be thin. She states that her eating disorder began by self-starvation in an attempt to be more pious, and she had hopes of becoming a Saint through asceticism. This unusual case is important to examine as we look at some of the core characteristics of AN, so that we can better understand and treat AN, a deadly illness with a high lifetime mortality rate [1].

## 2. Case Presentation

“Jane” is a 66-year-old woman who was admitted for treatment of AN that she reported to have begun when she was approximately 13-year old. Jane was raised in a suburb of Chicago and was the oldest of four with three younger brothers. She describes her childhood as happy and denies any history of abuse. She was raised by both her parents; her father was a lithographer and her mother was a home-maker. As the oldest child, Jane states that she was her mother's “little helper,” and she felt she wanted to do everything to please everybody. She reports having a positive relationship with her father and described him as her “best friend.” She felt that her mother was very busy raising her three younger brothers and felt she favored them over her. When Jane started kindergarten, she describes what may have been separation anxiety and would cry often for her mother. She describes herself as shy and had a few close friends who lived in her neighborhood. In elementary school, she reports being an anxious child; if a nun yelled at the whole class, she would cry. Jane did well in school, had excellent grades, and never missed more than 1-2 days of school.

Jane was raised catholic; her parents attended church every Sunday. When she was in the first grade, she was enrolled in a catholic school and was one of fifty students taught by a nun. In second grade, she began staying after school to help the nuns. One of the nuns took a special interest in her, and over time Jane decided she wanted to become a nun and ultimately a Saint. This never changed as she grew up, and her goal today is to become a Saint. Jane sang in choir and did her prayers excessively starting in 2nd grade. She is not sure how much time she spent on her rituals at the time, but as an adult, she spends over 2 hours per day in a ritualistic and obsessive manner, which included meditating.

Jane was not underweight growing up and states she did not worry about her weight in childhood. She began menarche at about the age of 12 and states she developed early for her age compared to the other girls. When she noticed her breasts developing, she brought it to the attention of her mother who told her “we do not talk like that.” She states that her eating disorder began when she fasted for lent at age of 13 and that she continued fasting after lent. She began losing weight and has had amenorrhea since the age of 13. At the age of 13, she joined the convent to become a nun where she continued to restrict her caloric intake. Joining the convent was what Jane perceived as her decision. In fact, she remembers her father was initially against her joining the convent but was told by their priest that if her father did not allow Jane to join the convent, then he was standing in the way of her occupation and he would go to hell. She left her family and moved several hours away, only seeing her family every other month during her adolescence.

At that convent, Jane lived in a dorm-room with about 20 girls, with curtains separating the cells. She states that it was strict; they could only talk at certain times, but she thought the convent was wonderful as she had friends with the same goals as hers. In the convent, Jane continued to restrict her caloric intake. Meals were served “family style,” and the girls were required to eat everything they took on their plate. Jane would take less food, though she does not recall how much she ate, and would finish everything on her plate. She recalls that the nuns would tell the girls that they could choose to make a sacrifice and take a smaller cookie or that they could be perfect and choose to go without a cookie, something Jane often did. She states that there were no full-length mirrors, and she only had a small mirror to put her habit on. There were also no scales and she is unaware of how much she weighed at the time.

During her adolescence, the convent underwent some major changes. Between about 1962 and 1965, the second Vatican council began addressing issues between the Roman Catholic Church and the modern world. Jane's supervisors began monitoring what she ate and asked her to see a psychiatrist. She did not understand why she needed to do this as she thought that she was being pious by restricting what she ate. At the age of 21, she left the convent as directed by her supervisors because of concerns about her low body weight. She was told that her practices were “too extreme” and it was “God's will” for her to leave the convent.

A few months after leaving the convent, Jane started nursing school and later married at the age of 25. She states that this was a culture shock for her as she lived in a co-ed dorm with pharmacy and medical students as well as nursing students. At the age of 21, she did not know even basic information about sexual intercourse and reproductive health. At this time Jane was very thin; she was 5 feet 0 inches and weighed about 75 pounds with a body mass index (BMI) of 14.6 kg/m^2^.

After she was married, she had difficulty conceiving and was seen by an endocrinologist who instructed her that if she gained weight, she may be able to have a baby. During this time, she tried eating more normally in an attempt to have a baby, and she reached her highest lifetime weight of 92 pounds (BMI of 18.0 kg/m^2^). She states that, for most of her life, she weighed less than 80 pounds (BMI of 15.6 kg/m^2^) which is considered to be severely underweight. She states that they later adopted three children and stopped trying to conceive after 7 years of infertility.

Jane was first treated for AN at the age of 30 years in 1976 and then not again until the age of 52 years in 1998. She has been previously treated 12 times in either residential or inpatient treatment facilities for eating disorder and had been working with an outpatient eating disorder treatment team that consisted of a psychiatrist, a nutritionist, and a therapist. She has had nasogastric (NG) tube placements multiple times and has tried multiple different psychotropic medications, including fluvoxamine 100 mg by mouth daily, started to treat comorbid obsessive compulsive disorder (OCD). She denies any history of binge-eating, laxative abuse, diuretic use, or diet pill use. She reports that, in addition to restricting her caloric intake, she exercises compulsively and does Pilates or aerobics 2 hours in the morning, about 3 hours of gardening, and 1-2 hours of walking around her house. Jane states that, in the past, she was able to recover “easily” from her low body weight, but as she has aged, she has had elevated liver function tests. Her comorbid medical conditions include gastroesophageal reflux, osteoporosis, macular degeneration, chronic obstructive pulmonary disease, and hypothyroidism. She denies any family psychiatric history. She denies an family psychiatric history or any personal history of alcohol or recreational drug use. She was working as a registered nurse up until her admission.

On admission, her weight was 60 pounds, her height was 4′10′′, and she had a BMI of 12.5 kg/m^2^. She had elevated AST of 460 U/L and elevated ALT of 745 U/L that resolved with treatment and weight restoration and was presumed to be secondary to malnutrition. She also had hyponatremia with sodium of 129 and leukopenia with a WBC of 2.3 mill/cu mm that also resolved. She was mildly anemic with hemoglobin of 11.6 g/dL and hematocrit of 24.5%, which also resolved with weight restoration. She reported some depression and grief since the passing of her husband 5 years ago. She also reported thoughts of wanting to be with her deceased husband in heaven, but denied any active suicidal thoughts due to her religious beliefs. She also endorsed OCD symptoms with compulsive religious rituals, compulsive exercising, and body checking. She stated that she still strives for Sainthood. She was treated in the inpatient eating disorder unit for a total of 50 days, and her weight improved to 80.8 lbs with a BMI 16.6 kg/m^2^.

During her treatment, Jane participated in group therapy that incorporated cognitive behavioral therapy (CBT), dialectical behavioral therapy (DBT), and expression therapy including art and yoga therapy. She also worked with an individual therapist and in family therapy that involved her adult adoptive children. In individual therapy, treatment included therapy that incorporated her spiritual beliefs while also challenging some of her religious beliefs (e.g., restriction as being a part of her eating disorder rather than part of her religious beliefs). Interestingly, as treatment progressed, Jane acknowledged that some component of her eating disorder did involve fat phobia and a desire to be thin and that she was not engaging in her behavior purely for religious reasons. She stated that the idea of weighing more than 80 pounds was “terrifying” to her. She had body image disturbance and would often pinch the skin around her abdomen to check for body fat. Over one year later since she left treatment, she continues to work with her outpatient treatment team, remains underweight, but has remained healthy enough to stay out of the hospital.

## 3. Discussion

Jane had no full-length mirrors or scales, did not know her weight, and did not count calories when she first developed her eating disorder. She states that she began losing weight to be more holy.

While this is unusual for contemporary women with AN, there have been multiple publications that indicate voluntary self-starvation found throughout history and in different sociocultural contexts [7–10]. In the Christian context, there are multiple examples of girls who fasted to the point of death and were even elevated to Sainthood. Termed anorexia mirabilis, this is different from anorexia nervosa in that anorexia mirabilis is associated with other ascetic practices such as flagellations and life-long virginity rather than starvation to achieve thinness, which is currently associated with modern AN [10]. Another main difference is that the behaviors of those with anorexia mirabilis were viewed at the time within a religious context and were not considered a disease [10].

One example of a girl (St. Wilgefortis) elevated to sainthood after self-starvation occurred sometime between 700 A.D. and 900 A.D. Her father, the King of Portugal, was going to force her to marry a suitor, though the girl had made a vow of virginity and service to God. Upon news of her engagement, she prayed that she be stripped of all her beauty and refused nourishment. She lost her feminine contours, and hair grew all over her body. Her suitor withdrew his offer of matrimony. The girl was crucified by her father in punishment. She became the Patron Saint for those who wish to be relieved from the problems associated with procreation or even those under control of others. With time, she became known as one who had liberated herself from the physical and social burdens of womankind [8].

Another example is of Saint Catherine of Siena who began lengthy meditations, reportedly cut her hair, ate very little, and forced herself to vomit the little she had ingested. She also flagellated herself in imitation of Christ's passion. She died at the age of 32 from malnutrition. She was a model for later religious fasters [9].

Between the 13th and 17th century, mostly in southern Europe, there were 181 cases of holy fasting described, and there are numerous examples of women fasting to the point of death. It was thought that they had direct communication with God in addition to this being a means of avoiding arranged marriages and childbirth [9].

It is interesting that so many of the holy fasters were young women and not men. Many of the women were elevated to a higher social status and even achieved fame, something that would not have occurred had they become wives and mothers. There is some discussion whether anorexia mirabilis represents a historical continuation of AN or whether it is an entirely separate entity. While it is unlikely that they are the same thing, AN and anorexia mirabilis certainly represent a long line of females who use their bodies and food as a symbolic language.

Miraculous fasting continued throughout the seventeenth and eighteenth centuries and cases of these “miraculous maids,” as they were called at the time, brought skepticism and wonder, and clergymen, physicians, and civil magistrates would conduct around-the-clock investigations, sometimes resulting in the death of the girls when the extreme fasting was taken too far [7, 10]. Often they came from poor families and became local attractions with visitors who brought gifts and money [7, 10].

Those who are unfamiliar with the history of AN may be surprised to learn that AN was first described in the 19th century prior to mass media and cultural pressures to diet and the perception that thin is beautiful. AN was first described in the late 19th century almost simultaneously by Leseque in France and Gull in England in 1873. [7] The young females were described as having a “delirious conviction that they cannot or ought not to eat” [7] and who presented with oppositional behaviors as well as an obsession with food [7]. Physicians were cautioned to remove the patient from her family for forced feeding. Both Gull and Leseque described, in addition to refusal to eat, onset in early adulthood or adolescence, restlessness, amenorrhea, and lack of concern from the part of the patient over her worsening condition. They also were rather optimistic regarding the prognosis if the patient is removed from the family who may interfere with treatment. The diagnosis of AN in the 19th century did not appear to include body image disturbance or fear of being fat [7].

The case of Jane is a hybrid between anorexia mirabilis and AN in the sense that, while occurring in modern time, her reasons for voluntary self-starvation and the cultural context in which she was raised are more similar to those individuals described in history as having anorexia mirabilis or miraculous maids. By examining some of the similarities and differences, one can understand more deeply the core criteria of AN. Jane's presentation is similar in many ways to modern AN. For one, she developed her eating disorder at the age of 13 during puberty, which is in the peak age of onset for AN [1]. She also is highly perfectionistic and has comorbid anxiety disorders including OCD, which has a higher prevalence rate in AN [1]. She also has had amenorrhea since the age of 13 and meets the DSM-5 criteria for AN (has persistent restriction of intake resulting in significantly low body weight, persistent behavior that interferes with weight gain, and persistent lack of recognition of seriousness of current low body weight). She also reported body image disturbance later on in treatment and was overly concerned about fat around her abdomen, despite being extremely thin and even expressed extreme concern and fear of weighing more than 80 pounds. She also had health consequences of osteoporosis and elevated liver function enzymes, which is seen in severe malnutrition.

There are some particularly unusual aspects of the case: one being that she was removed from the family environment from the age of 13 and had little contact with her parents after the age of 13. Another somewhat unusual aspect of this case is the age of the patient and her high functioning status despite being underweight for her entire adulthood. Jane worked as an RN for over 40 years, was married, and adopted 3 children that she helped to raise. While she had health consequences including osteoporosis and possibly liver changes as a result of malnutrition, overall, she appeared to be in relatively fair health, not having suffered as many health consequences as one might expect.

One must be careful not to state that fasting causes AN, similarly to the fact that dieting does not cause AN, as this does not appear to be the case. Still fasting may be a trigger or risk factor for AN, but this would need to be examined more carefully.

In some ways, her religious views both helped her in terms of her resilience as well as impeded in her journey to recovery. This case also shows the importance of examining the patient's perceived causes of the eating disorder. One important aspect for Jane is to examine and help her gain insight into which of her perceived religious beliefs actually have a spiritual basis and which are more based in her eating disorder mentality. For instance, during treatment, she was encouraged not to engage in excessive religious rituals in the morning, and she reduced the length of these rituals from 2 hours each day to 1 hour each day. She was encouraged to examine what purpose the rituals truly served her. Her treatment would for instance be very different from a patient who presented stating she had started having her eating disorder to achieve thinness or in order to be healthier.

While the reasons for triggering the disorder are different, there are strong similarities between them, certain core traits, such as a high degree of perfectionism, a need for self-control, and a lack of insight into the severity of their illness and/or the seriousness of their low body weight. While one should be careful not to apply a modern diagnosis such as AN to a historical context such as medieval Saints, one can see how an individual might have symptoms of an eating disorder that manifests similarly to the medieval Saints and in an attempt to emulate the Catholic Saints. In reviewing the historical perspective of AN, one also gains a better appreciation of how a mental illness can manifest itself differently in different cultural and historical contexts and yet the core symptomatology remains strikingly similar. This case, therefore, is an argument against that theory that AN is a “culturally bound disease,” a concept that, while refuted by psychiatrists, is still possibly misinterpreted by the public today. AN, a multifactorial disease, is influenced by genetic, biological, social, and cultural factors and is not solely a product of western culture. This unique case offers depth to our understanding of the development of AN and shows how multiple sociocultural influences can affect its development.
